# Time to update the Japanese standard population for comparing mortality rates

**DOI:** 10.1186/s13690-022-00908-0

**Published:** 2022-06-06

**Authors:** Bibha Dhungel, Koji Wada, Hirokazu Tanaka, Stuart Gilmour

**Affiliations:** 1 Graduate School of Public Health, St. Luke’s International University, Tokyo, 104-0045 Japan; 2 Department of Health Policy, National Centre for Child Health and Development, Tokyo, 157-0074 Japan; 3 Graduate School of Public Health, International University of Health and Welfare, Tokyo, 107-8402 Japan; 4 Division of Surveillance and Policy Evaluation, Institute for Cancer Control, National Cancer Center, Tokyo, 104-0045 Japan

**Keywords:** Standard population, Standardise, Mortality, Age standardisation, Japan, Trends

## Abstract

For the last three decades, Japan has been using the population of 1985 for age standardisation to compare mortality rates over time. With the population of Japan declining and ageing rapidly every year, there is a need to update the standard population to make the comparison representative of the current scenario. This is particularly relevant owing to declining mortality rates among the super-ageing Japanese elderly population and more data availability for older age groups. The choice of one population as standard over another is arbitrary because it does not make much difference to the trends in rates. The proportion of elderly in Japan is increasing rapidly and is expected to be one-third of the total population by 2030, in contrast to the proportion of 10% in the 1980s. Using a standard population with a lower proportion of elderly may weight the rates disproportionately for this age group. It is typically suitable to change the standard population every 25 to 30 years. It is advisable to choose the population of 2015 as the new standard population as suggested by the working group of the Ministry of Health, Labour and Welfare of Japan for revising the standard population. However, it should be noted that the newly calculated age-standardised mortality rates will no longer be comparable to those calculated using the older standard populations. Updating the standard population will produce age-standardised rates for recent years closer to the crude rates and would thus reduce the extent of misinterpreting decreased mortality risks using age-standardised rates that do not closely resemble the crude rates.

## Background

Incidence, prevalence, and mortality rates are common metrics used to evaluate the effectiveness of public health policies and community interventions. Their use for policy evaluation often involves comparing rates over time. However, in epidemiology, these measures are subject to an increase or decrease based on changes in the age distribution of the population. When comparing rates over time, or between two or more groups, the population is subject to change, often resulting in different age structures caused by changes in birth rate, death rate, or migration. Demographers have proposed the use of life-expectancy for comparing mortality rates as it is based only on age-specific rates and is not affected by age structure. However, life expectancy at birth does not reflect disease or outcome-specific rates. Hence, age-standardisation has widely been adopted for comparing the outcome-specific rates after age adjustment. Depending on the data availability and population structure two major methods of standardisation are commonly used–direct and indirect standardisation. Indirect standardisation is preferred in smaller populations with few numbers of cases in certain age groups or when only limited information is available such as the total number of cases or deaths. On the other hand, direct standardisation is used for large populations with known age-specific rates. It is commonly applied in epidemiology to strengthen the comparison of death, incidence, or prevalence rates between two or more populations or over a period. Direct standardisation compares the weighted average age-specific rates of the populations being compared, thereby removing the effect of age composition, and is interpreted as counterfactual rates.

The standardised mortality rates for two population groups (A and B) are given by

$$DS{R}_a=\sum {r}_{ia}\left(\frac{n_{is}}{\sum_i{n}_{is}}\right)$$

$$DS{R}_b=\sum {r}_{ib}\left(\frac{n_{is}}{\sum_i{n}_{is}}\right),$$

where *r*_*ia*_ and *r*_*ib*_ are the rates for the *i*^th^ age groups in populations A and B, respectively, and *n*_*is*_ is the number of people in the i^th^ age group of a chosen standard population.

When comparing two groups that are different in space (e.g., the mortality rate of the populations of two countries), the standard population is usually chosen as the combined population of the groups in question, or as a global standard, such as a global or regional population. For example, when comparing prefectures in Japan in the same year, the national population might be used as the standard population. When comparing one group over time, however, a specific year needs to be chosen as the standard population, and the rates in all other years must be standardised relative to this year. For example, to compare mortality rates over time in Japan, it is essential to choose a specific year to standardize against to ensure that the mortality rates are compared against a stable background age structure. Without such standardisation, estimates of trends can be misleading or even completely wrong, as has been seen with suicide mortality in Japan [1].

The Japan Ministry of Health, Labour and Welfare (MHLW) has long recommended using the 1985 population as the standard population for age standardisation when comparing populations over time. A working group has been set up by the Japanese MHLW in 2019 for the revision of the standard population, however, the suggested smoothed population of 2015 as standard population has not been implemented yet [2]. As the Japanese population is ageing, the age distribution has been shifting toward older ages. This shift will have an effect on overall mortality rates. The proportion of people over 65 years old is expected to rise from 28.4% in 2019 to around 33.3% in 2036 [3]. The current choice of the standard population of 1985 is about two generations older and is very different from the distribution of the current population. This leads to age-standardised rates that are completely different from crude rates across the recent time period. Mortality risks between populations with varying age structures could be compared using age-specific mortality rates. However, the large number of comparisons for each age group make the age-specific calculations cumbersome. Thus, with a super-ageing Japanese population, there is a need to revise the currently used standard population for comparing rates over time.

In this paper, we examine the difference in rates and trends in crude and age-standardised all-cause mortality rates in Japan from 1980 to 2015. We obtained five-yearly occupation-specific mortality data coinciding with the census years from the vital statistics registration of the MHLW [4]. We obtained census population data for every 5 years from the Statistics Bureau of Japan [5]. We calculated the age-standardised mortality rates over the years using the five-year age-specific population for every 5 years from 1980 to 2015 as the standard population. A linear regression analysis of the trend of the directly standardized rates was conducted with the interaction of time and standardisation year. Linear combination of the key variables estimated the change in five-yearly trends of mortality rates using a different standard population from 1980 to 2015.

### Standard population in other countries

The United States of America used the 1940 census population as standard until the late 1990s. After a report from two national workshops in 1991 and 1997, it adopted the projected 2000 population as its new standard. The World Health Organization adopted the European and world standards for comparing rates across countries in 2001 [6]. Australia used the most recent census population for the year ending in ‘1’ as the standard population, thereby changing the standard population every 10 years. However, in 2011, it revised this policy and recommended continuing using the 2001 population as standard. Because changing the standard population every 10 years resulted in negligible changes, Australia suggested changing it every 25 years. Similarly, in 2013, the European Union changed its standard population to the average population over 2011 to 2030, projected on the basis of 2010 populations. These countries updated their standard population despite the fact that this change had minimal effect on the overall trends in rates in the respective countries. Instances from these countries and organisations suggest that a population that best describes the distribution of the current population over a period of time should be chosen as the standard population.

### Effect on rates of using different standard populations

**Fig. 1:**
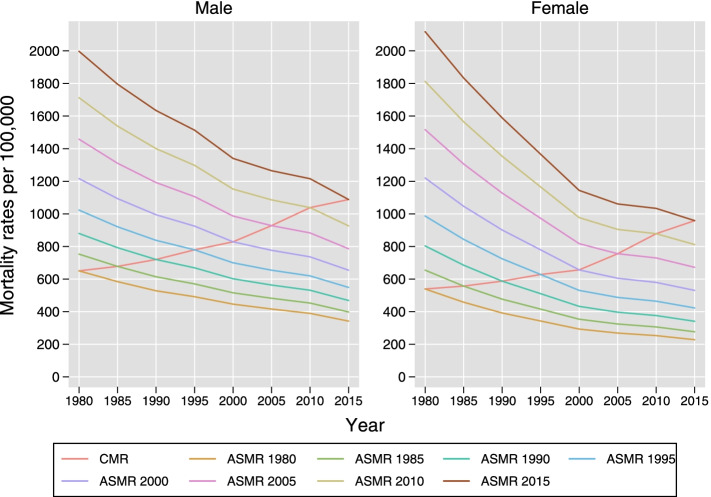
Trends in crude and age-standardised mortality rates per 100,000 separately by sex. Note: Rates for line plot ‘ASMR #’ is standardised using year #; CMR-Crude mortality rates; ASMR-Age-standardised mortality rates

Figure 1 shows the trends in crude and age-standardised all-cause mortality rates for all ages using each five-year population from 1980 to 2015 as the standard population. The absolute values of rates standardised using different population rates differ across the years. The age-standardised rates differ significantly from the crude rates; however, the trends in rates remain almost the same, although different in magnitude. Similarly, the estimated age-standardised rates using the recent population as standard would be closely approximate to the mortality risk in the current population as represented by the crude mortality rates.

Table 1 shows the crude and age-standardised mortality rates using a different standard population from 1980 to 2015. It also shows the overall percentage change from 1980 to 2015 in rates standardised using different standard populations. The overall percentage decline from 1980 to 2015 ranged from 45.5 to 47.4 among men and 54.7 to 57.7 among women.

**Table 1:** Crude and age-standardised mortality rates using different standard populations from 1980 to 2015

Directly standardised using population of year	Rates per 100,000					
	Men			Women		
	1980	2015	% change^**a**^	1980	2015	% change^**a**^
**Crude death rates**	650.3	1088.0	67.3	539.2	958.4	77.8
**1980**	650.3	341.9	−47.4	539.2	227.9	−57.7
**1985**	752.9	398.0	−47.1	654.7	276.5	−57.8
**1990**	879.7	468.5	− 46.7	803.4	340.8	−57.6
**1995**	1023.4	548.5	−46.4	986.7	422.7	−57.2
**2000**	1216.5	654.4	−46.2	1220.0	530.4	− 56.5
**2005**	1458.1	786.0	−46.1	1516.7	671.9	− 55.7
**2010**	1712.4	926.0	−45.9	1812.8	811.7	−55.2
**2015**	1997.3	1088.0	−45.5	2117.7	958.4	−54.7

^a^Absolute change in per cent from 1980 to 2015

Table 2 shows the relative change in age-standardised mortality rates from the crude rates in the same year. A very large relative difference in rates is evident from the table. Using more recent years as the standard population would largely overestimate the rates in previous years, specifically before 2000.

**Table 2:** Relative change in age-standardised mortality rates from the crude rates in the same year

Standardised using population of year	Per cent change in age-standardised rates from crude rates							
	1980	1985	1990	1995	2000	2005	2010	2015
**Men**﻿								
1980	0.0	−13.8	−26.7	−36.9	−46.2	−55.2	− 62.5	−68.6
1985	15.8	0.0	−14.7	−26.8	−37.8	−48.0	−56.4	− 63.4
1990	35.3	16.9	0.0	− 14.2	−27.4	−39.3	−48.8	−56.9
1995	57.4	35.9	16.3	0.0	−15.5	− 29.5	−40.3	− 49.6
2000	87.1	61.4	38.1	18.7	0.0	−16.3	−29.1	−39.9
2005	124.2	93.3	65.6	41.9	19.1	0.0	−14.9	−27.8
2010	163.3	127.0	94.5	66.5	39.1	17.0	0.0	−14.9
2015	207.1	164.9	126.9	94.2	61.8	36.3	17.1	0.0
**Women**								
1980	0.0	−17.8	−33.3	−45.4	−55.3	−64.4	−71.2	−76.2
1985	21.4	0.0	−18.8	− 33.8	−46.1	−57.1	− 65.1	− 71.2
1990	49.0	23.0	0.0	−18.7	− 34.1	−47.5	− 57.2	−64.4
1995	83.0	51.5	23.4	0.0	−19.2	− 35.5	−47.1	−55.9
2000	126.3	87.9	53.5	24.1	0.0	−20.0	−34.0	−44.7
2005	181.3	134.5	92.1	54.9	24.5	0.0	−16.9	−29.9
2010	236.2	181.0	130.5	85.5	48.9	19.7	0.0	−15.3
2015	292.8	229.3	170.7	117.5	74.3	40.4	17.7	0.0

Table 3 shows the change in trends of age-standardised mortality rates for different standard populations. The trend in age-standardised mortality rates decreases on average by 9.7 units (95% confidence interval (CI), − 11.8 to − 7.6) among men and by 10.5 units (95% CI, − 15.3 to − 5.7) among women every five-year when standardised using 1985 population. When standardised using the 2015 population, the trend in age-standardised rates decreases on average by 25.1 units (95% CI, − 27.2 to − 23.0) among men and by 33.1 units (95% CI, − 37.9 to − 28.4) among women for every increase in 5 years.

**Table 3:** Change in trends of age-standardised mortality rates from 1980 to 2015 for different standard populations

Standardised using population of year	Coefficient	95% Lower confidence interval	95% Upper confidence interval	*p*-value
**Men**				
1980	−8.4	−10.5	−6.3	< 0.001
1985	−9.7	−11.8	−7.6	< 0.001
1990	−11.2	−13.3	−9.1	< 0.001
1995	−13.0	−15.1	−10.9	< 0.001
2000	−15.4	−17.5	− 13.3	< 0.001
2005	−18.5	−20.6	−16.3	< 0.001
2010	−21.7	−23.8	−19.5	< 0.001
2015	−25.1	−27.2	− 23.0	< 0.001
**Women**				
1980	−8.62	−13.4	−3.8	< 0.001
1985	−10.5	−15.3	−5.7	< 0.001
1990	−12.9	−17.7	−8.2	< 0.001
1995	−15.8	−20.6	−11.1	< 0.001
2000	−19.5	−24.2	−14.7	< 0.001
2005	−24.0	−28.7	−19.2	< 0.001
2010	−28.5	−33.3	−23.7	< 0.001
2015	−33.1	−37.9	−28.4	< 0.001

### Choosing a standard population: discussion and recommendations

A key step in the process of standardisation is the choice of the standard population. This choice not only affects the age-standardised values but also the comparability between populations. It will help to closely estimate the mortality inequality across subgroups of populations such as the race and ethnic differentials in mortality [7]; however, it has very little impact on the trend in rates over time. When comparing rates over time, if the age-specific rates are consistent over the period being considered, the chosen standard population is of little consequence to the final conclusion. However, in the case of an inconsistent relationship between the age-specific rates in the population over the years, that is, increasing rates over the years in one age group (e.g. 0–5-year-olds) and decreasing rates over the years in the other age group (e.g. 80–85-year-olds), the comparison would differ depending on the choice of the standard population [7]. In such cases, the age-standardised death rates may mask the underlying trends in the age-specific rates. Thus, it is essential to examine both age-standardised, and age-specific rates to understand the underlying complexities in age-specific trends that may not be well reflected by standardisation.

The age-standardised mortality rates based on the current recommended standard population is lower than the rates calculated using the standard population from more recent years with a higher proportion of the elderly population. There is no conceptual justification for choosing a standard population for comparing rates. The age structure of the new standard population should be chosen to better reflect the age composition of the future population with which the rates will be compared and to provide numerically similar estimates of rates in recent years. A standard population with a similar age structure to the populations it is being compared with is preferred. Irrespective of which standard population is chosen, it should be noted that the age structure of the population will change over time. Thus, attempting to choose a standard population with a similar age structure to the current population is pointless. However, keeping in mind, the changing structure of the population, it is advisable to base the standard on the population that is similar to the age structure of the populations to be compared over the likely period that the new standard population will be used. Additionally, standardising the recent rates using the 1985 population is likely to underestimate the magnitude of change in rates over time as seen in F﻿ig. 1 and Table 3, thus underestimating the efforts of the government and the local bodies in reducing the mortality rates in recent years. Thus, as the new standard population is expected to be used for the next 25–30 years, it is advisable to consider the smoothed population of 2015 as standard as suggested by the study group of the MHLW for the revision of the standard population [2].

Even though WHO suggested changing the standard population to the projected 2000–2025 populations [6], many renowned organizations including the International Agency for Research on Cancer [8] has still been using the Segi’s world population devised in 1960 [9] as standard due to the unavailability of historical data for the newer age-categories. Bray et al. suggested that it is not necessary to replace the Segi world population for comparing cancer rates as they did not find much difference in the approximated relative risks [10]. Although the MHLW of Japan suggested additional age categories in the newly suggested standard population, it has not provided any corresponding data for the previous years. The unavailability of historical data for the age 0-year-olds and five-year age group data for those aged 85+ makes it difficult to implement the newly suggested standard population for comparing rates over time. Hence, even though more people are expected to enter the older age category, it might be better to keep the age categories as currently being used to avoid the fundamental difficulties while changing the standard population. The newer categories can be implemented for comparing rates over time if the MHLW provides relevant historical data for the age group 0, 1–4, 85–90, 90–95 and 95+ years. However, it should be noted that applying the new standard population would impose a huge cost on the national, prefectural, and municipality-level government.

Changing the standard population every few years is tedious because the historical rates need to be changed every time to make it comparable. Countries such as the United Kingdom and the United States of America have an arbitrarily chosen standard population and have used it over a period of time. Thus, we suggest changing the standard population again in Japan in 2045–2050. Changing a standard population will significantly affect the values of age-standardised rates. It must be carefully assessed before drawing conclusions. Using a recent population as standard will remarkably increase the resulting age-standardised rates over the years. Thus, it would be necessary to recalculate the age-standardised rates for previous years using the new standard population to make it comparable to recent years. To facilitate comparisons, the MHLW could provide data for the recalculated rates for all previous years. It would be interesting to measure the magnitude of the change of rates by cause of death; however, it should be noted that these changes do not reflect the risk of mortality from that cause. Updating the standard population will produce age-standardised rates for recent years closer to the crude rates and would thus reduce the extent of misinterpreting decreased mortality risks using age-standardised rates that do not closely resemble the crude rates.

## Data Availability

The data that support the findings of this study are available from the Japanese Ministry of Health, Labour and Welfare but restrictions apply to the availability of these data and so are not publicly available.
